# A Perspective on the Interaction Between Recurrent Lower Urinary Tract Infections and Irritable Bowel Syndrome

**DOI:** 10.3390/nu16213613

**Published:** 2024-10-24

**Authors:** Florina Ruța, Calin Avram, Elena Mardale, Mirela Pribac, Sara Suciu, Victoria Nyulas

**Affiliations:** 1 Department of Community Nutrition and Food Safety, George Emil Palade University of Medicine, Pharmacy, Science and Technology of Targu Mures, 540139 Targu Mureș, Romania; florina.ruta@umfst.ro; 2 Department of Medical Informatics and Biostatistics, George Emil Palade University of Medicine, Pharmacy, Science and Technology of Targu Mures, 540139 Targu Mureș, Romania; victoria.rus@umfst.ro; 3 Regina Maria—Policlinica Centrul Civic, 500173 Brașov, Romania; 4 Doctoral School of Biomedical Sciences, University of Oradea, 410073 Oradea, Romania; pribac.mirelaioana@student.uoradea.ro; 5 George Emil Palade University of Medicine, Pharmacy, Science and Technology of Targu Mures, 540139 Targu Mureș, Romania; suciu.sara-maria.21@stud.umfst.ro

**Keywords:** urinary infections, irritable bowel syndrome, dysbiosis, eating behavior and lifestyle

## Abstract

Introduction: Given the potential overlap in risk factors associated with both irritable bowel syndrome (IBS) and lower urinary tract infections (LUTIs), we aimed to identify factors that may contribute to the development of both conditions, as well as recurrent lower urinary tract infections (RLUTIs). Our research also sought to explore overlapping symptoms and interactions between these two disorders. Materials and Methods: The study included young women with a history of urinary tract infections. Participants were divided into three groups: women with sporadic LUTIs (NRLUTIs), women with recurrent LUTIs (RLUTIs), and women with both a history of urinary infections (NRLUTI or RLUTI) and a diagnosis of IBS. The diagnosis of IBS is primarily clinical, relying on symptoms and the exclusion of other gastrointestinal disorders. Data from intestinal microbiota tests were combined with information on patients’ symptom perception, dietary habits, lifestyle, and knowledge regarding their conditions. Results: Abdominal pain, constipation, insufficient knowledge about antibiotic and probiotic use, and nutritionally unbalanced diets were identified as common factors associated with both LUTI-IBS and RLUTI. Conclusions: Our research identified shared risk factors between LUTI, IBS, and RLUTI, suggesting a pathological interdependence between these conditions. Notably, women with RLUTIs often experience gastrointestinal symptoms such as abdominal pain and constipation after consuming foods known to trigger IBS. This highlights that gut dysbiosis is both a risk factor and a potential consequence of RLUTI. The presence of either condition appears to exacerbate the symptoms of the other, further underscoring the intricate connection between RLUTI and IBS in affected individuals.

## 1. Introduction

Studies have shown that women with irritable bowel syndrome (IBS) have a similar risk of lower urinary tract infections (LUTIs) as healthy women, despite experiencing more intense LUTI symptoms, regardless of IBS subtype or severity [1]. IBS is characterized by a range of symptoms, most notably abdominal pain and changes in bowel habits [2,3]. Although IBS itself does not directly cause urinary problems, many IBS triggers including stress and infection [4] are thought to potentially contribute to urinary issues. Common bladder symptoms in individuals with IBS include frequent urination, incomplete bladder emptying, nocturia, and urinary urgency. Women with IBS may also have a higher likelihood of urinary incontinence and an overactive bladder (OAB) compared to women without IBS [5].

A 2012 study conducted by the University of Medicine Asahikawa in Japan found that 33.3% of individuals with IBS also exhibited general hyperactive behavior [4]. It is unclear whether IBS predisposes individuals to hyperactivity of certain organs such as an overactive bladder, urinary tract problems or vice versa. In some cases, treating one condition can lead to improvement in the other [4]. Given the anatomical proximity of the intestine and bladder, a possible neuromuscular interaction between the two systems may exist. Alternatively, causes such as inflammation near kidneys and intestines, or a neurological issue affecting the entire region, could play a role in the symptomatology of both conditions [4,6,7]. However, these conditions significantly impact quality of life and there is high need to understand interdependency in order to develop new therapeutic strategies.

The aim of the present study was to identify new potential risk factors for both lower urinary tract infections (LUTI) and irritable bowel syndrome (IBS), as well as to explore possible overlaps in their manifestations and interactions between the two disorders.

## 2. Materials and Methods

### 2.1. Inclusion Criteria

The subjects of the study were selected exclusively from female patients with documented lower urinary tract infections (LUTIs) at family practice clinics, outpatient care clinics, and the dietetics clinic in the Mureș region of Romania. The age range for inclusion in the study was 18–45 years old.

Women diagnosed with LUTI were selected based on the following criteria: those who experienced ≤3 symptomatic episodes of LUTI in the past year or 2 episodes in the last 6 months, classified as recurrent urinary tract infection (RLUTI), and those who had ≤1 episode of LUTI in the past 12 months without recurrence, classified as nonrecurrent urinary tract infection (NRLUTI). Additionally, women with sporadic LUTI episodes over the past five years were included. LUTI was defined by a uroculture showing ≥10^3^ CFU/mL of uropathogenic bacteria. All selected patients had at least one episode of LUTI confirmed by ultrasound. Results regarding antibiotic resistance were based on laboratory assessments of microorganism susceptibility. Women with irritable bowel syndrome (IBS) among the selected participants were also identified by collaborating medical staff after clinical evaluation.

Collecting statistics: The study participants completed a questionnaire to evaluate their history of LUTI (including disease and treatments), the information they received from medical staff regarding LUTI, as well as their dietary behavior and lifestyle. Participants were able to select responses based on the frequency of LUTI episodes and the symptoms they experienced. The level of knowledge about LUTI was measured on a 1–5 scale, where levels 1–2 indicated “none or little information”, level 3 indicated “some information”, and levels 4–5 indicated “enough or a lot of information”. Regarding food consumption and eating behavior, the questionnaire allowed participants to select symptoms related to food intake. Eating behavior was also assessed on a 1–5 scale (1–2 for “rarely or never”, 3 for “sometimes”, and 4–5 for “usually or often”). For the quantitative evaluation of food intake, a shorter version of the Rapid Eating Assessment for Participants (REAP) model [8] was used.

All patients underwent a microbiota stool test at a specialized laboratory, which included a fecal culture that quantified levels of both putrefactive and protective bacteria. This stool analysis specifically measured the presence of potentially pathogenic bacteria, including *Escherichia coli*, *Klebsiella* spp., *Pseudomonas* spp., *Proteus* spp., *Enterobacter* spp., and *Clostridium* spp., among others, along with fungi such as *Candida* spp. Simultaneously, levels of beneficial bacteria, such as *Lactobacillus* spp., *Bifidobacterium* spp., and *Enterococcus* spp., were assessed. The results were reported in colony-forming units (CFUs), and the overall dysbiosis was graded on a scale: 1–5 indicated mild dysbiosis, 6–12 represented intermediate dysbiosis, and values over 12 signified pronounced dysbiosis. A single collection device was used to obtain 1 g of the biological sample. The sample was collected at least 7 days after completing any antibiotic treatment. Processing occurred within 5 days, the stability period for the biological sample, as long as it was consistently stored at a temperature between 2–8 °C. Dysbiosis was considered present if the flora index was ≥6.

Patients with other pathologies, and pregnant and lactating patients were excluded.

The diagnosis of IBS was established clinically by the medical evaluator and considered a medication-free condition.

Patients with urinary infections but without IBS were classified as LUTI-NIBS, while those with urinary infections and a diagnosis of IBS were classified as LUTI-IBS.

Informed consent was obtained from the women who agreed to participate in the study, along with the administration of the questionnaire. The questionnaire was completed with the assistance of trained medical personnel (medical assistants, dietitians), without including any personal identification data.

The study was carried out between November 2022–November 2023.

### 2.2. Statistical Data Analysis

The database was created in Microsoft Excel 2010 and statistical analysis was performed in IBM SPSS Statistics v. 22. For variables with numerical data, we calculated the mean and SD, and for dichotomous variables, we identified their number and percentage. The numerical data were checked for normality using the Shapiro–Wilk test and depending on this result we applied parametric or non-parametric tests. To the numerical data, we applied the *t*-test to identify statistical significance and for the association between the rest of the variables we applied the chi square test. We also calculated a logistic regression to identify associations between patients with LUTI-IBS and those with RLUTI; recurrence of urinary infections was associated with the presence of IBS along with LUTI, and low acidification flora was associated with both LUTI_IBS and RLUTI. The set confidence threshold was 95% (*p* < 0.05).

## 3. Results

A total of 167 participants were enrolled in the study, with an average age of 37.17 ± 8.82 years (Table 1). Of these, 42.51% (*n* = 71) had IBS, while 57.48% (*n* = 96) were not diagnosed with IBS (Table 2).

The majority of patients with LUTI-IBS (98.60%, *n* = 70) experienced at least one LUTI per year, and 93% (*n* = 66) had recurrent LUTIs (RLUTIs), showing a statistically significant association between LUTI-IBS and both urinary infection conditions. Recurrence of LUTIs two, three, or four times a year was more common in women with LUTI-IBS compared to those with LUTI-NIBS (19.70%, *n* = 14 vs. 5.20%, *n* = 5; 21.10%, *n* = 15 vs. 4.20%, *n* = 4; 11.30%, *n* = 8 vs. 5.20%, *n* = 5), with a statistically significant link between RLUTI frequency and IBS (Table 2). Abdominal pain and constipation were less pronounced in women with LUTI-NIBS, with significant differences in symptom expression between the two groups (Table 2). Additionally, poor information regarding the risks of repeated antibiotic treatments, the role of probiotics in LUTI management, and hygiene practices for LUTI prevention were also associated with LUTI-IBS (Table 2).

Unbalanced eating behavior was predominant in LUTI-IBS women through frequent consumption of coffee in the morning on an empty stomach, frequent consumption of fast food, consumption of salty and fatty snacks, processed sweets, consumption of sweets in the morning on an empty stomach, sweet drinks, and alcohol (Table 3).

A total of 90.10% (*n* = 60) of women with LUTI-IBS exhibited dysbiosis, defined as any score above 0 on the dysbiosis scale. Among these, 81.70% (*n* = 58) had elevated levels of putrefactive intestinal bacteria, with histamine-producing bacteria exceeding normal limits in 80.3% (*n* = 57) of the respondents. Additionally, low levels of acidifying flora were observed in 87.30% (*n* = 62), and fungal flora levels were above normal in 62% (*n* = 44) of participants. These findings indicate a statistically significant imbalance associated with LUTI-IBS (Table 4).

A significant percentage of RLUTI women complained of abdominal pain and constipation (34.60%, *n* = 27 and 43.60%), *n* = 34. The percentage of women who received a lot of information about the risk of antibiotic treatments (39.30%, *n* = 35) and the benefits of probiotics (28.10%, *n* = 25) in LUTI was higher in the NRLUTI category (Table 5). Gastrointestinal symptoms following the consumption of foods considered triggers in IBS were associated with RLUTI, specifically abdominal pain (34.60%, *n* = 27) and constipation (43.60%, *n* = 34) (Table 5). Poor information regarding the risk of antibiotic treatment and the benefits of probiotics were associated with RLUTI (Table 5).

Unbalanced eating behavior was prevalent among RLUTI women through frequent consumption of fast food, salty and fatty snacks, processed sweets, consumption of sweets in the morning on an empty stomach, sweet drinks, and alcohol (Table 6).

Dysbiosis was identified in 83.3% (*n* = 65) of women with RLUTI, while among those with NRLUTI it was present in 32.6% (*n* = 29). The association of RLUTI and dysbiosis was statistically significant, with a similar situation for putrefactive flora, histamine-producing flora, acidifying flora, and fungi (Table 7).

Recurrence of urinary infections was associated with the presence of IBS along with LUTI, and low acidification flora was associated with both LUTI_IBS and RLUTI (Table 8).

**Figure 1:**
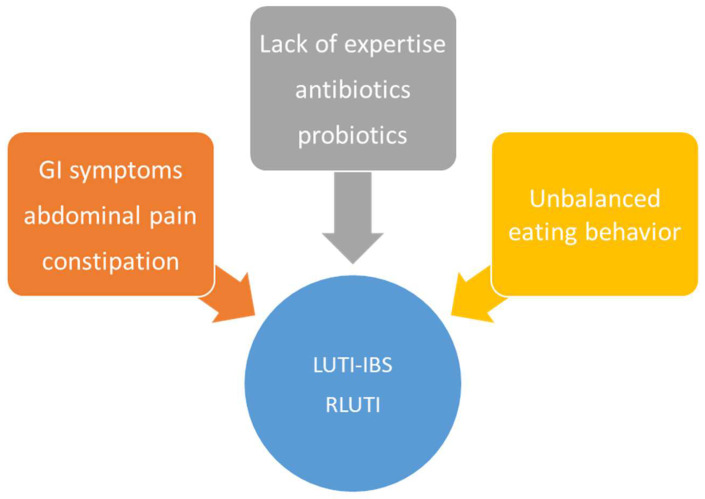
Common risk factors for LUTI-IBS and RLUTI.

Lower urinary tract infections and irritable bowel syndrome often have similar symp-toms and an interdependence between the two conditions is possible in that the pres-ence of one can exacerbate or worsen the symptoms of the other. Modifiable risk fac-tors related to diet and lifestyle are more prevalent among women with a history of both UTI and IBS (Figure 1).

## 4. Discussion

### 4.1. Irritable Bowel Syndrome in the Context of LUTI

Intestinal dysbiosis refers to an imbalance in the composition of gut microbiota, which can lead to various health issues, including gastrointestinal disorders and urinary tract infections. Recent studies have demonstrated that patients with IBS frequently exhibit gut dysbiosis, characterized by altered microbial diversity and composition [9]. Research indicates a reduction in beneficial bacteria, such as *Bifidobacterium* spp. and *Lactobacillus* spp., alongside an increase in potentially pathogenic species, including *Escherichia coli* and *Clostridium* spp. [10]. This dysbiotic state of the gut milieu is associated with symptoms such as abdominal pain, bloating, and altered bowel habits, suggesting that dysbiosis may contribute to the pathophysiology of IBS. The literature highlights that intestinal dysbiosis can also result from factors such as poor diet, antibiotic use, and lifestyle choices, contributing to conditions like IBS and recurrent urinary tract infections.

Numerous risk factors have been identified for the development of recurrent LUTI, a condition that significantly reduces quality of life [11]. One of the earliest studies to explore the connection between IBS and urinary tract infections was conducted by Whorwell et al. [12]. Following this, several epidemiological studies were performed to investigate the relationship between IBS and LUTI. Additionally, a meta-analysis of observational studies assessed the impact of IBS on LUTI-related symptoms in both men and women [13,14,15]. Matsumoto’s Japanese study of 10,000 participants found that both men and women with IBS were more likely to experience overactive bladder (OAB) [13]. In contrast to young women diagnosed with IBS alongside a history of LUTI, but otherwise healthy, our study focused on symptoms reported by patients, their level of knowledge about LUTI, relevant dietary and lifestyle behaviors, and symptoms associated with food consumption in a group of young women with LUTI-NIBS. Constipation and abdominal pain, which are common symptoms of LUTI [16], were less frequent in the LUTI-NIBS group. A Taiwanese study of 107 participants revealed that LUTI symptoms were more prevalent and severe in IBS patients [17]. Regarding knowledge about the impact of antibiotics on beneficial bacteria (risk of dysbiosis) and dietary recommendations for urinary infections, there were no significant differences between the two groups, with the rate of high knowledge being 29.20% vs. 29.60% and 17.70% vs. 28.20%, respectively, showing no statistically significant differences.

Gastrointestinal symptoms such as bloating, constipation, flatulence, abdominal pain, and nausea after consuming foods known to trigger GI symptoms [18] were predominantly observed in LUTI-IBS patients. The highest percentages were 46.50% for onions, garlic, and leek, and 22.50% for wheat-based products, while 36.50% of LUTI-NIBS patients did not experience adverse effects from these trigger foods.

Dietary behaviors considered risky, based on healthy eating guidelines from the literature [19], also highlighted differences between the groups. Coffee consumption on an empty stomach was more frequent in LUTI-IBS patients (54.90%) compared to LUTI-NIBS patients (36.50%). Studies have shown that higher caffeine intake is associated with an increased risk of developing IBS [20] and may also reduce UTI symptoms [21].

The profile of an unbalanced diet characterized by excessive consumption of processed foods (such as fast food), a low intake of vegetables (with the exception of potatoes), suboptimal consumption of fresh fruits, frequent intake of salty and fatty snacks, and a high frequency of processed desserts (including cakes, cookies, biscuits, wafers, pastries, doughnuts, muffins, chocolate, candies, and ice cream) significantly characterized the group of women with LUTI-IBS (Table 2). In both groups, the consumption of whole grains was similar, exceeding 35%, while the intake of salty foods was low in both groups, under 8%. Additionally, 10.40% of LUTI-NIBS women and 21.10% of LUTI-IBS women reported consuming less than 1 L of water per day.

Despite the lack of significant differences between the groups, approximately 25% of women did not consume enough dietary fiber, and about 20% did not drink enough water. Regarding alcohol consumption, 69.80% of LUTI-NIBS participants reported not drinking at all, compared to 47.90% of LUTI-IBS participants.

### 4.2. Recurrences of Lower Urinary Tract Infections

Most research on the risk factors for UTIs has focused on conditions such as diabetes, immunosuppressive medication use, and urinary catheterization, which are associated with an increased risk of UTIs [22]. In contrast, the impact of healthcare providers’ ability to effectively communicate information regarding risk factors and therapeutic measures—including antibiotic therapy, dietary habits, hygiene, lifestyle, and preventive behaviors—has been less extensively studied. *Escherichia coli*, responsible for 65–75% of urinary infections, can be found in ready-to-eat chicken breast prepared using sous-vide processing [23].

Foods that are spicy or acidic, along with caffeine, alcohol, high-sugar foods, or artificial sweeteners, may promote bacterial overgrowth and exacerbate symptoms by irritating the urinary tract. Sugar not only disrupts the intestinal microbiome but also suppresses immune system function, thereby increasing the risk of urinary infections and their recurrence [24]. The pH of the urinary tract, which influences the overgrowth of uropathogens [25], can be affected by dietary choices. A diet rich in animal proteins and excessive sodium intake lowers urinary pH, whereas a diet abundant in fruits and vegetables, along with adequate water intake and calcium-rich foods, contributes to a higher pH [26].

Supplementing the urinary tract with beneficial bacteria through probiotics may help prevent UTIs by restoring the natural balance of the intestinal bacterial flora, which affects both the urinary tract and the digestive system due to their close anatomical and functional connection [26]. Fruits, particularly berries rich in vitamins and antioxidants, can prevent bacteria from adhering to the walls of the urinary bladder [26]. Hydration is one of the most effective strategies for reducing the incidence of UTIs [27,28]. Additionally, omega-3 supplementation alongside standard medical therapy for urinary disorders has been shown to improve therapeutic outcomes [29,30]. A study by Gan et al. also indicated that low serum vitamin D levels are associated with an increased risk of UTI in a female pediatric population [31,32].

The lack of accurate information regarding LUTI, the risk of gut dysbiosis, and dietary recommendations did not differ between women with non-recurrent LUTI (NRLUTI) and those with recurrent LUTI (RLUTI). In both groups, nearly one-third of the women reported receiving insufficient information from medical staff.

Reduced consumption of whole grains was observed in one-third of all participants, while over half reported consuming semi-prepared foods, with no significant differences between the groups. Water consumption levels were similar across both groups; however, approximately 20% of respondents reported drinking less than one liter of water per day.

In addition to the well-known shared features of LUTI and IBS—such as their impact on quality of life and prevalence among young women, even in the absence of other underlying conditions—our study highlights additional common risk factors. These include more intense abdominal pain, constipation, lack of disease-related information, poor dietary habits, and alcohol consumption. Each of these factors was found at a higher rate in the more severe manifestations of these conditions, particularly among women diagnosed with both LUTI and IBS, as well as those experiencing frequent recurrences of lower urinary tract infections.

Addressing risk factors through hygienic and behavioral education, eliminating food triggers identified from nutritional labels [33,34], using dietary supplements, and avoiding unhealthy habits such as alcohol consumption can help mitigate complications associated with these conditions that have a significant negative impact on quality of life. The presence of gut dysbiosis and imbalances in intestinal microorganisms, which are strongly linked to both LUTI-IBS and RLUTI, further emphasizes the reversible nature of these risk factors. It is well established that dietary behavior significantly influences the intestinal microbiota [35,36,37,38,39].

The coexistence of IBS with LUTI, combined with intestinal dysbiosis, can elevate the risk of RLUTI. Furthermore, intestinal dysbiosis—characterized by an increase in putrefactive flora and a decrease in acidification—has been associated with both LUTI-IBS and RLUTI.

When addressing the treatment strategies for recurrent lower urinary tract infections (RLUTIs) and irritable bowel syndrome (IBS), the use of formal models can aid in simplifying intricate clinical decisions. Implementing such models allows healthcare professionals to make well-informed, consistent choices that take into account patient history and the variability in symptom patterns, which may lead to improved outcomes for individuals dealing with both RUTIs and IBS [40,41].

## 5. Conclusions

Through our study we have shown that uncomplicated diseases of the urinary and gastrointestinal tract, namely lower urinary tract infections and irritable bowel syndrome, often present with similar symptoms. Women with a history of UTIs who are also diagnosed with IBS tend to experience these symptoms more frequently and with greater intensity. In addition, modifiable risk factors related to diet and lifestyle are more prevalent among women with a history of both UTI and IBS, suggesting that people who are better informed about these factors have a lower risk of recurrent UTIs, IBS, and associated symptoms.

Our research also indicates a pathological interdependence between these two conditions, where the presence of one can exacerbate or worsen the symptoms of the other.

In conclusion, any strategy aimed at preventing these conditions—which have a significant impact on quality of life—should adopt a comprehensive approach that includes lifestyle, nutrition, education, and hygiene. As a distinct and complementary service to other medical treatments, healthcare providers must ensure that individuals are equipped with the necessary knowledge to adopt healthier personal and social behaviors with positive effects on both individual and community health.

## Figures and Tables

**Table 1:** Average age per study group.

*n* = 167	LUTI-IBS *n* = 71	LUTI-NIBS *n* = 96	*p* Value	RLUTI *n* = 78	NRLUTI *n* = 89	*p* Value
Age Mean (SD)	38.49 ± 8.46	36.19 ± 8.99	0.09	38.21 ± 8.46	36.25 ± 9.07	0.89

**Table 2:** Risk factors associated with LUTI-IBS vs. LUTI-NIBS.

Variables		Patients		*p* Value
		LUTI-NIBS *n* = 96	LUTI-IBS *n* = 71	
At least one LUTI/year	yes	18 (18.80%)	70 (98.60%)	0.0001
	no	78 (81.20%)	1 (1.40%)	
RLUTI	yes	12 (12.50%)	66 (93.00%)	0.0001
	no	84 (87.50%)	5 (7.00%)	
Frequency of LUTI/year	once	52 (54.20%)	25 (35.20%)	0.0001
	twice	5 (5.20%)	14 (19.70%)	
	three times	4 (4.20%)	15 (21.10%)	
	four times	5 (5.20%)	8 (11.30%)	
	five times	0	3 (4.20%)	
	six times	0	2 (2.80%)	
	more than six times	3 (3.10%)	3 (4.20%)	
	none reported	27 (28.10%)	1 (1.40%)	
Symptoms reported	abdominal pain	15 (15.60%)	22 (31.00%)	0.0001
	constipation	25 (26.00%)	32 (45.10%)	
	frequent diarrhea	5 (5.20%)	3 (4.20%)	
	alternating periods of diarrhea and constipation	7 (7.30%)	5 (7.00%)	
	none reported	44 (45.80%)	9 (12.70%)	
Accurate information about LUTI from the family doctor	not at all	29 (30.20%)	19 (26.80%)	0.49
	little	20 (20.80%)	12 (16.90%)	
	some	24 (25.00%)	14 (19.70%)	
	enough	11 (11.50%)	14 (19.70%)	
	a lot	12 (12.50%)	12 (16.90%)	
Information about the risk of antibiotic treatments	not at all	22 (22.90%)	30 (42.30%)	0.008
	little	12 (12.50%)	13 (18.30%)	
	some	11 (11.50%)	10 (14.10%)	
	enough	16 (16.70%)	6 (8.50%)	
	a lot	35 (36.50%)	12 (16.90%)	
Information about the role of probiotics in the treatment of LUTI	not at all	27 (28.10%)	38 (53.50%)	0.0001
	little	9 (9.40%)	12 (16.90%)	
	some	22 (22.90%)	13 (18.30%)	
	enough	12 (12.50%)	6 (8.50%)	
	a lot	26 (27.10%)	2 (2.80%)	
Information regarding the risk of intestinal dysbiosis	not at all	26 (27.10%)	21 (29.60%)	0.98
	little	11 (11.50%)	8 (11.30%)	
	some	18 (18.80%)	11 (15.50%)	
	enough	13 (13.50%)	10 (14.10%)	
	a lot	28 (29.20%)	21 (29.60%)	
Information about hygiene rules for the prevention of LUTI	not at all	23 (24.00%)	12 (16.90%)	0.02
	little	6 (6.20%)	17 (23.90%)	
	some	20 (20.80%)	13 (18.30%)	
	enough	12 (12.50%)	9 (12.70%)	
	a lot	35 (36.50%)	20 (28.20%)	
Information about appropriate dietary recommendations in LUTI	not at all	34 (35.40%)	21 (29.60%)	0.61
	little	18 (18.80%)	11 (15.50%)	
	some	20 (20.80%)	14 (19.70%)	
	enough	7 (7.30%)	5 (7.00%)	
	a lot	17 (17.70%)	20 (28.20%)	
Gastrointestinal symptoms after consumption of certain foods	onion, garlic, leek, cauliflower, mushrooms	10 (10.40%)	33 (46.50%)	0.001
	vegetables	16 (16.70%)	10 (14.10%)	
	dairy	9 (9.40%)	8 (11.30%)	
	fruits	11 (11.50%)	3 (4.20%)	
	wheat-based products	15 (15.60%)	16 (22.50%)	
	none reported	35 (36.50%)	1 (1.40%)	

**Table 3:** Food consumption associated with LUTI-IBS vs. LUTI-NIBS.

Variables		Patients		*p* Value
		LUTI-NIBS *n* = 96	LUTI-IBS *n* = 71	
Coffee in the morning on an empty stomach	never	42 (43.80%)	15 (21.10%)	0.03
	rarely	9 (9.40%)	6 (8.50%)	
	sometimes	5 (5.20%)	6 (8.50%)	
	usually	5 (5.20%)	5 (7.00%)	
	often	35 (36.50%)	39 (54.90%)	
Fast food twice a week or more	never	72 (75.00%)	38 (53.50%)	0.01
	rarely	8 (8.30%)	4 (5.60%)	
	sometimes	8 (8.30%)	7 (9.90%)	
	usually	5 (5.20%)	1 (1.40%)	
	often	3 (3.10%)	21 (29.60%)	
Less than 3 servings of vegetables (except potatoes)/day	never	28 (29.20%)	31 (43.70%)	0.03
	rarely	16 (16.70%)	14 (19.70%)	
	sometimes	24 (25.00%)	19 (26.80%)	
	usually	10 (10.40%)	4 (5.60%)	
	often	18 (18.80%)	3 (4.20%)	
Less than 2 servings of fruit per day	never	25 (26.00%)	29 (40.80%)	0.07
	rarely	24 (25.00%)	18 (25.40%)	
	sometimes	20 (20.80%)	16 (22.50%)	
	usually	7 (7.30%)	3 (4.20%)	
	often	20 (20.80%)	5 (7.00%)	
Less than 3 servings of whole grains/day	never	34 (35.40%)	25 (35.20%)	0.23
	rarely	20 (20.80%)	20 (28.20%)	
	sometimes	20 (20.80%)	19 (26.80%)	
	usually	7 (7.30%)	2 (2.80%)	
	often	15 (15.60%)	5 (7.00%)	
Snacks high in salt and fat	never	56 (58.30%)	23 (32.40%)	0.001
	rarely	15 (15.60%)	8 (11.30%)	
	sometimes	15 (15.60%)	10 (14.10%)	
	usually	4 (4.20%)	10 (14.10%)	
	often	6 (6.20%)	20 (28.20%)	
Processed sweets	never	27 (28.10%)	9 (12.70%)	0.04
	rarely	16 (16.70%)	14 (19.70%)	
	sometimes	23 (24.00%)	12 (16.90%)	
	usually	11 (11.50%)	13 (18.30%)	
	often	19 (19.80%)	23 (32.40%)	
Sweet food in the morning on an empty stomach	never	69 (71.90%)	38 (53.50%)	0.03
	rarely	12 (12.50%)	10 (14.10%)	
	sometimes	9 (9.40%)	3 (4.20%)	
	usually	2 (2.10%)	7 (9.90%)	
	often	4 (4.20%)	13 (18.30%)	
Semi-prepared foods	never	52 (54.20%)	40 (56.30%)	0.28
	rarely	18 (18.80%)	17 (23.90%)	
	sometimes	16 (16.70%)	10 (14.10%)	
	usually	2 (2.10%)	3 (4.20%)	
	often	8 (8.30%)	1 (1.40%)	
Sweet juices or more than 150 mL of freshly squeezed fruit juice	never	57 (59.40%)	30 (42.30%)	0.001
	rarely	15 (15.60%)	4 (5.60%)	
	sometimes	13 (13.50%)	9 (12.70%)	
	usually	3 (3.10%)	3 (4.20%)	
	often	8 (8.30%)	25 (35.20%)	
Less than 1 L of water per day	never	49 (51.00%)	34 (47.90%)	0.11
	rarely	7 (7.30%)	7 (9.90%)	
	sometimes	19 (19.80%)	13 (18.30%)	
	usually	11 (11.50%)	2 (2.80%)	
	often	10 (10.40%)	15 (21.10%)	
Alcohol	never	67 (69.80%)	34 (47.90%)	0.06
	rarely	14 (14.60%)	15 (21.10%)	
	sometimes	9 (9.40%)	12 (16.90%)	
	usually	3 (3.10%)	4 (5.60%)	
	often	3 (3.10%)	6 (8.50%)	

**Table 4:** Intestinal bacterial composition in LUTI-NIBS vs. LUTI-IBS.

Variables		Irritable Bowel Syndrome		*p* Value
		LUTI-NIBS *n* = 96	LUTI-IBS *n* = 71	
Dysbiosis	yes	30 (31.20%)	64 (90.10%)	0.0001
	no	66 (68.80%)	7 (9.90%)	
The flora of rot	increased	50 (52.10%)	58 (81.70%)	0.0001
	normal	46 (47.90%)	13 (18.30%)	
Histamine-producing flora	increased	44 (45.80%)	57 (80.30%)	0.0001
	normal	52 (54.20%)	14 (19.70%)	
Acidifying flora	normal	51 (53.10%)	9 (12.70%)	0.0001
	low	45 (46.90%)	62 (87.30%)	
Fungi	increased	29 (30.20%)	44 (62.00%)	0.0001
	normal	67 (69.80%)	27 (38.00%)	

**Table 5:** Risk factors associated with NRLUTI vs. RLUTI.

Variables		Patients		*p* Value
		NRLUTI *n* = 89	RLUTI *n* = 78	
Symptoms reported	abdominal pain	10 (11.20%)	27 (34.60%)	0.0001
	frequent constipation	23 (25.80%)	34 (43.60%)	
	frequent diarrhea	5 (5.60%)	3 (3.80%)	
	none reported	45 (50.60%)	8 (10.30%)	
	alternating diarrhea/constipation	6 (6.70%)	6 (7.70%)	
Accurate information about LUTI from the family doctor	not at all	28 (31.50%)	20 (25.60%)	0.61
	little	17 (19.10%)	15 (19.20%)	
	some	22 (24.70%)	16 (20.50%)	
	enough	10 (11.20%)	15 (19.20%)	
	a lot	12 (13.50%)	12 (15.40%)	
Information about the risk of antibiotic treatments	not at all	20 (22.50%)	32 (41.00%)	0.003
	little	10 (11.20%)	15 (19.20%)	
	some	10 (11.20%)	11 (14.10%)	
	enough	14 (15.70%)	8 (10.30%)	
	a lot	35 (39.30%)	12 (15.40%)	
Information about the role of probiotics in the treatment of LUTI	not at all	26 (29.20%)	39 (50.00%)	0.0001
	little	7 (7.90%)	14 (17.90%)	
	some	21 (23.60%)	14 (17.90%)	
	enough	10 (11.20%)	8 (10.30%)	
	a lot	25 (28.10%)	3 (3.80%)	
Information regarding the risk of dysbiosis	not at all	24 (27.00%)	23 (29.50%)	0.94
	little	9 (10.10%)	10 (12.80%)	
	some	17 (19.10%)	12 (15.40%)	
	enough	12 (13.50%)	11 (14.10%)	
	a lot	27 (30.30%)	22 (28.20%)	
Information about hygiene rules for the prevention of LUTI	not at all	21 (23.60%)	14 (17.90%)	0.08
	little	4 (4.50%)	19 (24.40%)	
	some	19 (21.30%)	14 (17.90%)	
	enough	12 (13.50%)	9 (11.50%)	
	a lot	33 (37.10%)	22 (28.20%)	
Information about appropriate dietary recommendations in LUTI	not at all	29 (32.60%)	26 (33.30%)	0.98
	little	15 (16.90%)	14 (17.90%)	
	some	19 (21.30%)	15 (19.20%)	
	enough	7 (7.90%)	5 (6.40%)	
	a lot	19 (21.30%)	18 (23.10%)	
Gastrointestinal symptoms after consumption of certain foods	onion, garlic leek, cauliflower, mushrooms	12 (13.50%)	31 (39.70%)	0.001
	vegetables	15 (16.90%)	11 (14.10%)	
	dairy	9 (10.10%)	8 (10.30%)	
	apples, pears, watermelon, dried fruits, hard pit fruits	10 (11.20%)	4 (5.10%)	
	products based on wheat and rye	12 (13.50%)	19 (24.40%)	
	none reported	31 (34.80%)	5 (6.40%)	

**Table 6:** Food consumption associated with NRLUTI vs. RLUTI.

Variables		Patients		*p* Value
		NRLUTI *n* = 89	RLUTI *n* = 78	
Coffee in the morning on an empty stomach	never	36 (40.40%)	21 (26.90%)	0.14
	rarely	9 (10.10%)	6 (7.70%)	
	sometimes	5 (5.60%)	6 (7.70%)	
	usually	7 (7.90%)	3 (3.80%)	
	often	32 (36.00%)	42 (53.80%)	
Fast food twice a week or more	never	66 (74.20%)	44 (56.40%)	0.001
	rarely	8 (9.00%)	4 (5.10%)	
	sometimes	8 (9.00%)	7 (9.00%)	
	usually	4 (4.50%)	2 (2.60%)	
	often	3 (3.40%)	21 (26.90%)	
Less than 3 servings of vegetables (except potatoes) per day	never	23 (25.80%)	36 (46.20%)	0.02
	rarely	17 (19.10%)	13 (16.70%)	
	sometimes	21 (23.60%)	22 (28.20%)	
	usually	8 (11.20%)	4 (5.10%)	
	often	18 (20.20%)	3 (3.80%)	
Less than 2 servings of fruit per day	never	22 (24.70%)	32 (41.00%)	0.07
	rarely	52 (25.80%)	19 (24.40%)	
	sometimes	21 (23.60%)	15 (19.20%)	
	usually	6 (6.70%)	4 (5.10%)	
	often	17 (19.10%)	8 (10.30%)	
Less than 3 servings of whole grains/day	never	3 (34.80%)	28 (35.90%)	0.59
	rarely	17 (19.10%)	23 (29.50%)	
	sometimes	19 (21.30%)	20 (25.60%)	
	usually	8 (9.00%)	1 (1.30%)	
	often	14 (15.70%)	6 (7.70%)	
Snacks high in salt and fat	never	55 (61.80%)	24 (30.80%)	0.001
	rarely	13 (14.60%)	10 (12.80%)	
	sometimes	11 (12.40%)	14 (17.90%)	
	usually	4 (4.50%)	10 (12.80%)	
	often	6 (6.70%)	20 (25.60%)	
Processed sweets	never	13 (11.50%)	9 (11.50%)	0.01
	rarely	18 (20.50%)	16 (20.50%)	
	sometimes	16 (17.90%)	14 (17.90%)	
	usually	10 (11.20%)	14 (17.90%)	
	often	17 (19.10%)	25 (32.10%)	
Sweet food in the morning on an empty stomach	never	64 (71.90%)	43 (55.10%)	0.04
	rarely	11 (14.60%)	9 (11.50%)	
	sometimes	7 (7.90%)	5 (6.40%)	
	usually	1 (1.10%)	8 (10.30%)	
	often	4 (4.50%)	13 (16.70%)	
Semi-prepared foods	never	49 (55.10%)	43 (55.10%)	0.60
	rarely	18 (20.20%)	17 (21.80%)	
	sometimes	12 (13.50%)	14 (17.90%)	
	usually	3 (3.40%)	2 (2.60%)	
	often	7 (7.90%)	2 (2.60%)	
Sweet juices or more than 150 mL of freshly squeezed fruit juice	never	55 (61.80%)	32 (41.00%)	0.005
	rarely	11 (12.40%)	8 (10.30%)	
	sometimes	12 (13.50%)	10 (12.80%)	
	usually	3 (3.40%)	3 (3.80%)	
	often	8 (9.00%)	25 (32.10%)	
Less than 1 L of water per day	never	43 (48.30%)	40 (51.30%)	0.29
	rarely	6 (6.70%)	8 (10.30%)	
	sometimes	19 (21.30%)	13 (16.70%)	
	usually	10 (11.20%)	3 (3.80%)	
	often	11 (12.40%)	14 (17.90%)	
Alcohol	never	5 (73.00%)	36 (46.20%)	0.008
	rarely	12 (13.50%)	17 (21.80%)	
	sometimes	8 (9.00%)	13 (16.70%)	
	usually	2 (2.20%)	5 (6.40%)	
	often	2 (2.20%)	7 (9.00%)	

**Table 7:** Intestinal microflora in NRLUTI vs. RLUTI.

Variables		Irritable Bowel Syndrome		*p* Value
		NRLUTI *n* = 89	RLUTI *n* = 78	
Dysbiosis	yes	29 (32.6%)	65 (83.3%)	0.0001
	no	60 (67.4%)	13 (16.7%)	
Putrefactive flora	increased	51 (57.3%)	57 (73.1%)	0.02
	normal	38 (42.7%)	21 (26.9%)	
Histamine-producing flora	increased	43 (48.3%)	58 (74.4%)	0.0001
	normal	46 (51.7%)	20 (25.6%)	
Acidifying flora	normal	45 (50.6%)	15 (19.2%)	0.0001
	low	44 (49.4%)	63 (80.8%)	
Fungi	increased	29 (32.6%)	44 (56.4%)	0.02
	normal	60 (67.4%)	34 (43.6%)	

**Table 8:** Factors associated with LUTI-IBS and RLUTI.

Variables	RLUTI			LUTI-IBS		
	*p*	OR	95% C.I	*p*	OR	95% C.I
LUTI-IBS	<0.0001	208.742	36.031–1209.334	-	-	-
Increased histamine-producing flora	0.045	17.817	1.069–296.828	0.319	0.331	0.038–2.919
Dysbiosis	<0.0001	12.127	4.767–30.847	0.014	6.849	1.478–31.737
Low acidification flora	0.001	4.029	1.715–9.464	<0.0001	8.211	3.091–21.813
Putrefactive flora	0.37	0.88	0.09–0.867	0.037	10.646	1.152–98.396
RLUTI present	-	-	-	0.001	33.989	4.425–261.065

## Data Availability

The data that support the findings of this study are available from the corresponding author upon reasonable request.
